#  Re-evaluating perioperative and neoadjuvant immunotherapy in early-stage lung cancer: Current evidence and discussions 

**DOI:** 10.5578/tt.2025011040

**Published:** 2025-03-24

**Authors:** Enes ERUL

**Affiliations:** 1 Division of Medical Oncology, Department of Internal Medicine, Ankara University Faculty of Medicine, Ankara, Türkiye

## Abstract

**ABSTRACT**

** Re-evaluating perioperative and neoadjuvant immunotherapy in early-stage lung cancer: Current evidence and discussions Hairy vocal cords and hemoptysis **

* Early-stage lung cancer remains a challenging disease with a significant risk of recurrence despite treatment. In recent years, there has been growing interest in the application of neoadjuvant and perioperative immunotherapies. The success of immune checkpoint inhibitors in advanced stages has prompted their investigation in earlier disease stages. This editorial examines clinical trials comparing the efficacy of perioperative and neoadjuvant immunotherapies, focusing on their impact on survival, pathological response rates, and toxicity profiles. Furthermore, ongoing debates and the importance of patient-centered decision-making are discussed.
*

**Key words:** *Early-stage lung cancer; neoadjuvant immunotherapy; perioperative treatment; immune checkpoint inhibitors; non-small cell lung cancer *

**ÖZ**

** Erken evre akciğer kanserinde perioperatif ve neoadjuvan immünoterapinin yeniden değerlendirilmesi: Mevcut kanıtlar ve tartışmalar **

* Erken evre akciğer kanseri, tedaviye rağmen tekrarlama riski taşıyan karma- şık bir hastalıktır. Son yıllarda neoadjuvan ve perioperatif immünoterapilerin kullanımına yönelik ilgi artmıştır. İmmün kontrol noktası inhibitörlerinin ileri evre hastalıklardaki başarısı, bu tedavilerin daha erken evrelerde uygulanabilirliğini gündeme getirmiştir. Bu editoryal, perioperatif ve neoadjuvan immünoterapilerin etkinliğini karşılaştıran klinik çalışmaları; bu yaklaşımların sağkalım, patolojik yanıt oranları ve toksisite profilleri üzerindeki etkilerini incelemektedir. Ayrıca tedaviye ilişkin mevcut tartışmalar ile hasta merkezli karar verme sürecinin önemi ele alınmaktadır. *

**Anahtar kelimeler:** *Erken evre akciğer kanseri; neoadjuvan immünoterapi; perioperatif tedavi; İmmün kontrol noktası inhibitörleri; küçük hücreli olmayan akciğer kanseri *

## Re-evaluating perioperative and neoadjuvant immunotherapy in early-stage lung cancer

Lung cancer remains the leading cause of cancer- related mortality in both men and women. However, three-year survival rates have improved from 22% for patients diagnosed between 2004 and 2006 to 33% for those diagnosed between 2016 and 2018. This notable improvement is likely due to advancements in surgical techniques, the development of medical therapies such as immunotherapy and targeted treat- ments, and the increased detection of early-stage lung cancer through enhanced screening programs (1). The introduction of immune checkpoint inhibitors, par- ticularly monoclonal antibodies targeting PD-1, PD-L1, and CTLA-4, has fundamentally transformed treatment algorithms for non-small cell lung cancer (NSCLC). Progression-free survival and overall sur- vival (OS) benefits demonstrated in randomized con- trolled trials for advanced-stage NSCLC have shifted the focus towards exploring the use of immunothera- py in earlier stages of the disease (2-4). The occur- rence of recurrence even in early resectable stages of lung cancer highlights the unmet need for effective treatment strategies. The availability of numerous dis- tinct therapeutic scenarios has further fueled debates regarding the optimal treatment choice.

Previous neoadjuvant approaches using chemothera- py, such as preoperative administration of carbopl- atin and paclitaxel in early-stage cases, failed to demonstrate a significant difference in disease-free survival compared to postoperative adjuvant chemo- therapy (5). However, with the CheckMate 816 trial, the addition of three cycles of nivolumab to plati- num-based neoadjuvant chemotherapy introduced the concepts of major pathologic response, patho- logical complete response (pCR), and residual viable tumor (RVT) (6). The combination of three cycles of nivolumab and chemotherapy prior to surgery dem- onstrated a significant benefit compared to chemo- therapy alone, with a hazard ratio (HR) of 0.68 [95% confidence interval (CI), 0.48-0.93] for disease pro- gression or death. Based on the outcomes achieved with preoperative therapy, discussions have begun to focus on treatment escalation and de-escalation strat- egies, as well as the potential use of circulating tumor DNA for monitoring and guiding therapy (6,7). While OS is commonly used as an endpoint in oncology, its evaluation often requires longer follow-up. Therefore,

event-free survival (EFS) has been increasingly uti- lized in lung cancer studies. It is well-established that preoperative therapies achieving pCR and reduced RVT are associated with improved EFS outcomes (7).

A significant challenge in clinical trials evaluating neoadjuvant or perioperative (neoadjuvant-adjuvant immunotherapy) approaches lies in the heterogeneity of the study populations. This heterogeneity arises from variations such as the inclusion of different stages (ranging from IB to IIIA in some trials and II to III in others) and discrepancies in staging methods, with some studies using the tumor, node, metastasis (TNM) 7th edition and others employing the TNM 8th

edition (6,8-15) (Table 1). Unfortunately, the debate surrounding perioperative, neoadjuvant, or adjuvant treatment approaches continues to rely on indirect findings and comparisons. This is primarily due to the lack of a randomized, three-arm trial directly com- paring neoadjuvant immunotherapy combined with chemotherapy, neoadjuvant chemotherapy alone, and a perioperative approach within the same study.

The intact interaction between the primary tumor and its draining lymph nodes is thought to favor neoadju- vant immunotherapy strategies. This approach is sup- ported by the hypothesis that lymph node resection may limit the efficacy of immunotherapy by reducing the activation of anti-tumor T-cell responses (16). Another hypothesis favoring the neoadjuvant approach is the concern that adjuvant immunothera- py may increase toxicity. In an indirect meta-analysis including perioperative approaches from trials such as KEYNOTE-671, Neotorch, AEGEAN, and NADIM II, compared to chemotherapy alone, and CheckMate 816, which involved only neoadjuvant immunother- apy, no significant differences were observed in EFS (HR, 0.90; 95% CI, 0.63-1.30; p= 0.59) or OS (HR,

1.18; 95% CI, 0.73-1.90; p= 0.51). However, treat-

ment-related adverse events of any grade were sig- nificantly higher in the perioperative approach, where adjuvant immunotherapy was included (rela- tive risk, 1.08; 95% CI, 1.00-1.17; p= 0.04) (17).

An individual patient-level data analysis of CheckMate 77T and CheckMate 816 was conducted to assess the contribution of the adjuvant phase in the periopera- tive nivolumab treatment regimen. The analysis dem- onstrated a benefit in EFS [HR (95% CI): Unweighted,

0.59 (0.38-0.92)]. However, the carry-over effect of the initial neoadjuvant immunotherapy component

Tuberk Toraks 2025;73(1):80-84 **81**

**Table:** 

**Table 1.** Overview of clinical trials evaluating neoadjuvant, adjuvant and perioperative approaches in lung cancer								
**Trial**	**Timing**	**Size Agent I/O**	**Cycles, n**	**Inclusion**	**Stage IB+II/III,** **%**	**Primary endpoint**	**Chemotherapy**	**EGFR/ALK**
		1.005 Atezolizumab (PD-L1) 1.177 Pembrolizumab (PD-1) 1.415 Durvalumab (477) (PD-L1) (PD-1) (PD-L1) (PD-1) 797 Pembrolizumab (PD-1) (PD-1) 453 Tislelizumab (PD-1)		Completely				
IMpower0108	Adjuvant		16	resected IB (>4 cm)-IIIA	59/41	DFS hierarchical	Cisplatin doublet	Included (15%)
				(7th)				
				Completely				
KEYNOTE-0919	Adjuvant		18	resected IB (>4 cm)-IIIA	72/28	DFS, DFS in PD-L1 ≥50%	Platinum doublet encouraged	Included (7.4%)
				(7th)				
BR.3110	Adjuvant		12	Completely resected IB (>4 cm)-IIIA (7th) ESTS	71/39	DFS, PD-L1 ≥25%, EGFR/ ALK WT	Platinum doublet if not eligible	Included
				Resectable IB				No
CheckMate-8166	Neoadjuvant		3	(>4 cm)-IIIA	36/64	pCR, EFS	Platinum doublet	documented
				(7th)				mutation
				Resectable				
	Perioperative		16	II-IIIB (8th) by	29/71	pCR, EFS	Platinum based	WT: Asia
				lobectomy				
Neotorch12	Perioperative		17	Resectable II-IIIB (8th)	20/80	MPR, EFS	Platinum based	No documented mutation
KEYNOTE-67113	Perioperative		13	Resectable II-IIIB (8th)	30/70	EFS, OS	Cisplatin doublet	WT
	Perioperative		16	Resectable II-IIIB/ (8th)	35/65	EFS	Platinum doublet	No EGFR, no documented ALK
RATIONALE-31515	Perioperative		12	Resectable II-IIIA (8th)	41/59	EFS, MPR	Platinum doublet	WT
OS: Overall survival, EFS: Event-free survival, pCR: Pathological complete response, DFS: Disease-free survival, WT: Wild-type, PD-1: Programmed death-1, PD-L1: Programmed death- ligand 1, EGFR: Epidermal growth factor receptor, ALK: Anaplastic lymphoma kinase, MPR: Major pathologic response.								

Erul E.

cannot be entirely ruled out. Additionally, although PD-L1 is known to be an imperfect biomarker, the observed benefit was significant in PD-L1-negative patients [PD-L1 <1%, HR: 0.51 (0.28-0.93)] while showing overlap in PD-L1-positive patients [PD-L1

≥1%, HR: 0.86 (0.44-1.70)], continuing to raise ques- tions and uncertainties (18). Nonetheless, the strong- est hypothesis supporting the perioperative approach is that the KEYNOTE-671 trial, with its extended fol- low-up duration, is the only study demonstrating an OS benefit (13). In the perioperative study of pem- brolizumab, the KEYNOTE-671 trial, the relationship between pathological regression and EFS revealed that when more than 60% RVT remained, the pem- brolizumab and placebo arms showed overlapping results (19). This finding raises critical questions such as: Is postoperative adjuvant therapy truly necessary? Should we persist with adjuvant therapy in cases where neoadjuvant treatment has failed to elicit a response?

Amidst all these ongoing debates, a fundamental principle in oncology remains unchanged: Treatment selection should consider patient’s preference, finan- cial toxicity, shared decision-making, and drug avail- ability. Until more definitive evidence emerges, per- sonalized decision-making based on the patient’s preference will be the most appropriate approach.

## CONFLICT of INTEREST

The author have no conflict of interest to declare.

## AUTHORSHIP CONTRIBUTIONS

Concept/Design: EE Analysis/Interpretation: EE Data acqusition: EE Writing: EE

Clinical Revision: EE Final Approval: EE

## REFERENCES

1. *Howlader N, Forjaz G, Mooradian MJ, Meza R, Kong CY, Cronin KA, et al. The effect of advances in lung-cancer treatment on population mortality. N Engl J Med 2020; 383(7): 640-9. https://doi.org/10.1056/NEJMoa1916623*
2. *Hellmann MD, Paz-Ares L, Bernabe Caro R, Zurawski B, Kim SW, Carcereny Costa E, et al. Nivolumab plus ipilim- umab in advanced non-small-cell lung cancer. N Engl J Med 2019; 381(21): 2020-31. https://doi.org/10.1056/NEJMoa1910231*
3. *Sezer A, Kilickap S, Gümüş M, Bondarenko I, Özgüroğlu M, Gogishvili M, et al. Cemiplimab monotherapy for first- line treatment of advanced non-small-cell lung cancer with PD-L1 of at least 50%: A multicentre, open-label, global, phase 3, randomised, controlled trial. Lancet 2021; 397(10274): 592-604. https://doi.org/10.1016/S0140-6736(21)00228-2*
4. *Reck M, Rodríguez-Abreu D, Robinson AG, Hui R, Csőszi T, Fülöp A, et al. Five-year outcomes with pembrolizumab versus chemotherapy for metastatic non-small-cell lung cancer with PD-L1 tumor proportion score ≥ 50. J Clin Oncol 2021; 39(21): 2339-49. https://doi.org/10.1200/JCO.21.00174*
5. *Felip E, Rosell R, Maestre JA, Rodríguez-Paniagua JM, Morán T, Astudillo J, et al. Preoperative chemotherapy plus surgery versus surgery plus adjuvant chemotherapy versus surgery alone in early-stage non-small-cell lung cancer. J Clin Oncol 2010; 28(19): 3138-45. https://doi.org/10.1200/JCO.2009.27.6204*
6. *Forde PM, Spicer J, Lu S, Provencio M, Mitsudomi T, Awad MM, et al. Neoadjuvant nivolumab plus chemotherapy in resectable lung cancer. N Engl J Med 2022; 386(21): 1973-85. https://doi.org/10.1056/NEJMoa2202170*
7. *Deutsch JS, Cimino-Mathews A, Thompson E, Provencio M, Forde PM, Spicer J, et al. Association between patho- logic response and survival after neoadjuvant therapy in lung cancer. Nature Medicine 2024; 30(1): 218-28. https://doi.org/10.1038/s41591-023-02660-6*
8. * Felip E, Altorki N, Zhou C, Csőszi T, Vynnychenko I, Goloborodko O, et al. Adjuvant atezolizumab after adju- vant chemotherapy in resected stage IB-IIIA non-small-cell lung cancer (IMpower010): A randomised, multicentre, open-label, phase 3 trial. Lancet 2021; 398(10308): 1344-57. https://doi.org/10.1016/S0140-6736(21)02098-5*
9. *O’Brien M, Paz-Ares L, Marreaud S, Dafni U, Oselin K, Havel L, et al. Pembrolizumab versus placebo as adjuvant therapy for completely resected stage IB-IIIA non-small- cell lung cancer (PEARLS/KEYNOTE-091): An interim analysis of a randomised, triple-blind, phase 3 trial. Lancet Oncol 2022; 23(10): 1274-86. https://doi.org/10.1016/S1470-2045(22)00518-6*
10. *Goss G, Darling GE, Westeel V, Nakagawa K, Massuti Sureda B, Perrone F, et al. LBA48 CCTG BR.31: A global, double-blind placebo-controlled, randomized phase III study of adjuvant durvalumab in completely resected non-small cell lung cancer (NSCLC). Annals of Oncology 2024; 35: S1238. https://doi.org/10.1016/j.annonc.2024.08.2289*
11. *Heymach JV, Harpole D, Mitsudomi T, Taube JM, Galffy G, Hochmair M, et al. Perioperative durvalumab for resect- able non-small-cell lung cancer. N Engl J Med 2023; 389(18): 1672-84. https://doi.org/10.1056/NEJMoa2304875*
12. *Lu S, Zhang W, Wu L, Wang W, Zhang P; Neotorch Investigators. Perioperative toripalimab plus chemotherapy for patients with resectable non-small cell lung cancer: The neotorch randomized clinical trial. JAMA 2024; 331(3): 201-11. https://doi.org/10.1001/jama.2023.24735*
13. *Wakelee H, Liberman M, Kato T, Tsuboi M, Lee S-H, Gao S, et al. Perioperative pembrolizumab for early-stage non- small-cell lung cancer. New Eng J Med 2023; 389(6): 491-503. https://doi.org/10.1056/NEJMoa2302983*
14. *Cascone T, Awad MM, Spicer JD, He J, Lu S, Sepesi B, et al. Perioperative nivolumab in resectable lung cancer. N Engl J Med 2024; 390(19): 1756-69. https://doi.org/10.1056/NEJMoa2311926*
15. *Yue D, Wang W, Liu H, Chen Q, Chen C, Liu L, et al. Perioperative tislelizumab plus neoadjuvant chemothera- py for patients with resectable non-small-cell lung cancer (RATIONALE-315): An interim analysis of a randomised clinical trial. Lancet Respir Med 2024; S2213-2600(24): 00269-8.*
16. *Fear VS, Forbes CA, Neeve SA, Fisher SA, Chee J, Waithman J, et al. Tumour draining lymph node-generated CD8 T cells play a role in controlling lung metastases after a primary tumour is removed but not when adjuvant immunotherapy is used. Cancer Immunol Immunother 2021; 70(11): 3249-58. https://doi.org/10.1007/s00262-021-02934-3*
17. *Zhou Y, Li A, Yu H, Wang Y, Zhang X, Qiu H, et al. Neoadjuvant-adjuvant vs neoadjuvant-only PD-1 and PD-L1 inhibitors for patients with resectable NSCLC: An indirect meta-analysis. JAMA Netw Open 2024; 7(3): e241285-e. https://doi.org/10.1001/jamanetworkopen.2024.1285*
18. *Forde PM, Peters S, Donington J, Meadows-Shropshire S, Tran P, Lucherini S, et al. PL02.08 perioperative vs neoad- juvant nivolumab for resectable NSCLC: Patient-level data analysis of checkmate 77T vs checkMate 816. J Thorac Oncol 2024; 19(10): S2. https://doi.org/10.1016/j.jtho.2024.09.014*
19. * DR, Wakelee H, Spicer JD, Liberman M, Kato T, Tsuboi M, et al. OA01.03 association of pathologic regression with EFS in the KEYNOTE-671 study of perioperative pembrolizumab for early-stage NSCLC. J Thorac Oncol 2024; 19(10): S8. https://doi.org/10.1016/j.jtho.2024.09.022*

